# Treatment of Inherited Retinal Dystrophies with Somatic Cell Therapy Medicinal Product: A Review

**DOI:** 10.3390/life12050708

**Published:** 2022-05-09

**Authors:** Giacomo Maria Bacci, Valentina Becherucci, Elisa Marziali, Andrea Sodi, Franco Bambi, Roberto Caputo

**Affiliations:** 1 Pediatric Ophthalmology Unit, Children’s Hospital A. Meyer-University of Florence, 50139 Florence, Italy; elisa.marziali@meyer.it (E.M.); roberto.caputo@meyer.it (R.C.); 2 Cell Factory Meyer, Children’s Hospital A. Meyer-University of Florence, 50139 Florence, Italy; valentina.becherucci@meyer.it (V.B.); franco.bambi@meyer.it (F.B.); 3 Department of Neuroscience, Psychology, Drug Research and Child Health, University of Florence, 50139 Florence, Italy; andreasodi2@gmail.com

**Keywords:** inherited retinal dystrophies, Stargardt disease, retinitis pigmentosa, advanced therapy medicinal products, somatic cell therapy medicinal products, good manufacturing practices, retinal progenitor cells, induced pluripotent stem cells, embryonic stem cells, mesenchymal stromal cells, retinal organoids

## Abstract

Inherited retinal dystrophies and retinal degenerations related to more common diseases (i.e., age-related macular dystrophy) are a major issue and one of the main causes of low vision in pediatric and elderly age groups. Advancement and understanding in molecular biology and the possibilities raised by gene-editing techniques opened a new era for clinicians and patients due to feasible possibilities of treating disabling diseases and the reduction in their complications burden. The scope of this review is to focus on the state-of-the-art in somatic cell therapy medicinal products as the basis of new insights and possibilities to use this approach to treat rare eye diseases.

## 1. Introduction: Retinal Architecture and Inherited Retinal Dystrophies

### 1.1. Retinal Architecture Overview

The retina is a complex neural network with the main goal of transducing light through electrical pulse encoding and packing visual input for visual pathways and superior visual elaboration. This process requires a sequence of events where every cell plays a definite role in the visual signal processing [1]. The first actors in this process are retinal photoreceptor cells; essentially, these specialized, first-order neurons located in the outer retina have the structure and the metabolic privilege to convert light into electricity in photopic and scotopic conditions through a molecularly driven complex defined as the visual cycle. The electric pulse is then transmitted to the second-order neurons, the bipolar cells, and, subsequently, to the third-order neurons, the ganglion cells, forming the optic nerve core with their unmyelinated axons [2]. Photoreceptor, bipolar and ganglion cells represent the main pre-geniculate circuit for visual signaling transmission located in the eye, but ancillary cell subtypes, such as amacrine cells and horizontal cells, contribute to modulating this primary pathway [3]. The other main actor in retinal anatomy is the RPE, which represents a pillar to maintain the structural and functional role of the entire retina; its strict relationship with the underlying complex Bruch’s membrane/choroid and the overhanging outer retina makes it a fundamental monolayer of polarized cells for the metabolic function of the retina [4].

### 1.2. Inherited Retinal Dystrophies (IRDs)

IRDs are defined as a group of degenerative disorders of the retina with clinical and genetic heterogeneity. IRDs can occur from birth through late middle age, with symptoms including night blindness, visual field abnormalities, dyschromatopsia and various degrees of central visual acuity impairment. Genotype–phenotype correlations are achieved by using electrophysiology and sophisticated imaging modalities [5]. Thanks to the increasing knowledge regarding the genetic basis of these diseases, authorized gene-therapy programs are available [6]. Clinical manifestations of IRDs can span from challenging cases, difficult to diagnose because they can present only subtle retinal abnormalities, to devastating disorders where the retinal architecture is severely affected early in life. As a consequence of these large presentation differences, retinal morphologic appearance alone can often be misleading for diagnosis or, at least, not enough for posing a specific diagnosis [7]. Until recent years, no treatment was available for these potentially devastating disorders, and supportive treatment (i.e., with low vision aids), together with monitoring of the disease, was the only approach once molecular diagnosis and family segregation analysis was defined. In this era of molecular engineering, new treatment opportunities are arising, bringing hope to families and patients. In this review, we will mainly focus on the description of two main early-onset IRDs that have a high impact on retinal anatomy and present ideal targets for an ATMPs approach, namely, STGD1 and Retinitis RP [6].

### 1.3. Stargardt Disease (STGD1)

STGD1 is the more common cause of macular degeneration in children and young adults related to mutations in the ABCA4 gene [8]. The pattern inheritance is autosomal recessive, and its prevalence span is from 1:8000 to 1:10,000. Generally speaking, the disease onset could be considered a surrogate prognostic marker: the earlier one is the disease onset, while the more severe one could be the presentation and evolution of the phenotype. A detailed description of STGD1 is beyond the scope of this review, so we will focus on aspects consistent with the ATMPs approach. Usually, STGD1 patients present with disease onset at a median age of 15 years, with the classic phenotypic fishtail appearance of yellow-white flecks at the posterior pole, eventually associated with a “bull’s eye” maculopathy, and the best-corrected visual acuity can vary between 20/70 and 20/200 [9]. Complete phenotyping with OCT, FAF and ffERG is mandatory for clinical diagnosis, together with a blood sample for molecular analysis; evidence of a severely reduced macular thickness with a diffuse alteration of the ellipsoid zone in the macular area is a frequent finding in typical STGD1, while diffuse hypofluorescence at the posterior pole, hyperfluorescence of retinal flecks and sparing of peripapillary autofluorescence are frequent features; electrophysiology results are extremely variable and classification of disease stage with ffERG has been proposed, according to rod and cone involvement during the course of the disease. In the presence of a coherent phenotypic clinical picture, mutations in the ABCA4 gene usually confirm the diagnosis [10]. This can be extremely challenging since the previous typical description cannot be respected in early-onset disease or widespread forms of cone–rod dystrophies, which are a potential expression of ABCA4 mutations [10]. Because of its extremely variable clinical course, STGD1 is potentially a perfect target for a therapeutic approach using advanced therapy medicinal products along different stages of the disease. Briefly, in early-onset disease, the macular function can be variably affected, but the integrity of the retinal architecture can still be preserved, while in late-onset disease, a variable degree of retinal atrophy can be the presenting sign [11]. As a consequence of these anatomical presentations, a different approach can be useful: mainly supportive in early-onset disease and regenerative in the atrophic phase.

### 1.4. Retinitis Pigmentosa (RP)

RP is the most frequent IRD, affecting more than 1.5 million patients worldwide [12]. RP has a widely variable age of onset, from childhood to adulthood [13]. RP can be associated with extra-ocular abnormalities in the syndromic form of RP. The term RP encompasses a group of progressive IRDs characterized by the primary degeneration of rod photoreceptors, followed by the loss of cone photoreceptors. Normally, the first symptom is reduced night vision (nyctalopia), which is classically followed by a progressive loss of peripheral vision. The macula and, consequently, the visual acuity are usually relatively well-preserved at the onset and could be involved in the late stages of the disease. Sometimes, the visual acuity can be affected earlier in cases of cataracts or cystoid macular oedema, both common and treatable sequelae of retinitis pigmentosa [13]. The classical fundus presentation includes the triad: bone spicule pigmentation predominantly in the periphery and/or mid-periphery, waxy pallor of the optic nerve head and attenuation of retinal vessels. The bone spicules do not develop in all patients; in some patients, dust-like pigmentation or nummular hyperpigmentation is reported [14]. Bone spicule pigmentation consists of RPE cells that detach from the Bruch’s membrane following photoreceptor degeneration and migrate to intraretinal perivascular sites, where they form melanin pigment deposits. The clinical findings in RP vary widely due to the large number of genes involved. Mutations in more than 80 genes have been implicated in non-syndromic RP, and each year, new genes are added to this list. There are gene-specific subtypes of RP with a specific age of onset, visual impairment, retinal appearance and/or rate of progression. Moreover, several factors can vary widely within each of these gene-specific subtypes, even in the same family, suggesting the presence of unidentified genetic and/or environmental factors that can influence the RP phenotype [12]. Early-onset RP tends to progress more rapidly. In general, patients affected by X-linked RP (5–15% of RP patients) have a more severe disease progression compared to patients with autosomal recessive RP (50–60% of RP patients), while patients with an autosomal dominant form of RP (30–40% of RP patients) have the best long-term prognosis [13]. The rapidly increasing knowledge about gene-specific subtypes of RP has shifted attention to individual therapies. However, various treatment strategies are exploring the applications of gene- and cell-based therapies, retinal implants or transplantation. The specific genetic defect, the molecular pathogenesis and the extent of the degeneration determine which treatment will be the most suitable for each RP patient.

## 2. Somatic Cell Therapy Medicinal Products: Definition and Regulation

sCTMPs are defined as a group of ATMPs containing or consisting of cells or tissues that have been significantly manipulated to modify their biological characteristics, physiological functions or structural properties or that are not intended to be used for the same original functions in the body. The purpose of somatic cell therapy is to treat, prevent or diagnose diseases. The definition of sCTMPs is currently included in Directive 2009/120/EC amending Directive 2001/83/EC of the European Parliament and the Council on the European Community. An sCTMP is “a biological medicinal product whose active substance is made by a living organism. The product has the following characteristics: (a) contains or consists of cells or tissues that have been subject to substantial manipulation so that biological characteristics, physiological functions or structural properties relevant for the intended clinical use have been altered, or of cells or tissues that are not intended to be used for the same essential function(s) in the recipient and the donor; (b) is presented as having properties for, or is used in or administered to human beings to treat, prevent or diagnose a disease through the pharmacological, immunological or metabolic action of its cells or tissues”. The cells or tissues can be of autologous (derived from the patient himself), allogeneic (obtained from a donor) or xenogeneic (derived from a donor of an animal species other than man) origin [15]. The safety and efficacy of a somatic cell therapy medicinal product must be demonstrated through preclinical studies and human clinical trials. Human clinical trials must be particularly designed in compliance with EU regulation No. 536/2014, following the principles of GCPs as reported in Commission Directive 2005/28/EC [16,17]. The production of a CTMP must be carried out following the principles of GMP. The particularity of ATMPs consists in their extreme complexity, which makes them unique and distinguishable from other medicinal products, starting from their composition and all the necessary processes for their proper development (i.e., manufacturing, characterization). During the last few years, the development of ATMPs for the treatment of eye diseases has become a fast-growing field. The field of ATMPs is currently at the forefront of innovation as it offers novel therapeutic approaches for the treatment of pathologies that, at present, have limited or no effective alternatives. For several reasons, the eye is an ideal organ for the application of ATMPs. First, it has small dimensions, thus requiring low amounts of medicinal products for treatment. Second, the anatomical structure is compartmentalized, thus limiting the distribution of medicinal products to non-target tissues. Third, it has good accessibility for applying treatments and examining outcomes. Fourth, it is isolated from the rest of the body due to the blood–retinal barrier. This makes the eyeball an immunologically privileged site because it restricts the passage of immunoglobulins. These reasons are why ATMPs present a great potential to improve the prognosis of and potentially cure ocular diseases that currently have no effective treatment, such as inherited retinal dystrophies.

## 3. Excursus on Somatic Cell Therapy Medicinal Products for Inherited Retinal Dystrophies

Currently, a high percentage of the clinical trials that are being carried out to study the efficacy and/or safety of sCTMPs for eye diseases are focused on the analysis of cell-based medicinal products [18]. In contrast to gene therapy, the number of authorized sCMTPs is lower, probably due to the technical difficulty and the high costs involved in developing a cell-based therapy and proving its safety and efficacy [19]. At the same time, there are four kinds of therapeutic approaches to using sCTMPs for IRDs [20] dealing with stem cells: RPCs, ESCs, iPSCs and MSCs [21]. The replacement of cells that are lost via transplantation represents one possible strategy for the treatment of inherited retinal dystrophies. One of the challenges in this approach is to identify and characterize sources and types of cells for transplantation [22,23]. Several cell populations may be identified as potential sources for retinal transplantation. These populations include RPCs, adult stem cells such as MSCs, ESCs and iPSCs. Characteristics of each cell type and their mechanisms of action are briefly described below.

### 3.1. Retinal Progenitor Cells (RPCs)

RPCs derived from fetal or neonatal retinas comprise a population of immature cells that are responsible for the generation of all retinal cells during development. Immature RPCs can be extensively expanded in vitro by manipulating time and environment and lead to the expression of photoreceptor markers [24]. Retinal repair by the transplantation of photoreceptor precursors, such as RPCs from the developing retina, into the dystrophic mature retina has been found to promote the survival of host tissue, along with integration into the neural retina and recovery of light-mediated behavior [25,26]. Unfortunately, these early studies of photoreceptor transplantation had limited success due to the poor durability of the integration into the recipient retina. Moreover, the degenerating retina is characterized by a hostile environment to the transplanted cells and strongly restricts the ability of the cells to migrate from the transplantation site into the host retina [27]. Recently, several publications reported the integration of transplanted photoreceptor precursors into the advanced degenerated retina. It has also been shown that the outcome of rod-photoreceptor precursor transplantation depends on different types and stages of degeneration [28]. According to these studies, functional rod-photoreceptor transplantation can be achieved by tailored manipulations of the host environment and appropriate therapeutic time windows [29]. On the other hand, since the protocol involves harvesting RPCs from fetal eyes, it cannot be translated to human patients due to ethical issues [30].

### 3.2. Embryonic Stem Cells (ESCs)

Currently, cell replacement therapy for the treatment of retinal diseases focuses on the development of protocols for the direct differentiation of hESCs or hiPSCs into RPCs and photoreceptor cell phenotypes. ESCs are derived from the inner cell mass of the embryonic blastocyst, with self-renewal capabilities and the ability to differentiate into cell types derived from all three embryonic germ layers [31,32]. In vitro differentiation of mouse and human ESCs into different functional retinal cell types, in particular, RPE cells and/or photoreceptors, has been demonstrated by numerous protocols [33,34]. Moreover, several studies have shown that the transplantation of ESCs derived from different species of retinal cells, in models of retinal degeneration, protected host photoreceptors, integrated into the recipient retina, differentiated into functional photoreceptors and restored visual function [35,36]. Thus, the transplantation of photoreceptors with or without RPE cells derived from hESCs offers huge potential for cell replacement therapy in treating retinal degenerative diseases [37]. Clinical trials in the United States using human ESC-derived RPE to treat Stargardt disease and AMD were approved by the FDA [38]. Furthermore, mouse ESCs can be induced to generate an eye-like structure made up of lens cells, retinal cells and RPE cells [39], and it has subsequently been shown that cells from these eye-like structures can be differentiated into RGCs when transplanted into the vitreous body of an injured adult mouse retina [40]. Recently, the therapeutic potential of ESCs has been extensively studied as they can be induced to aggregate and organize into a stratified optic cup in a three-dimensional (3D) culture system [41] and into a reconstitution of 3D retinal tissue in vitro [42,43]. The futuristic study by Schwartz et al. successfully transplanted ESC-derived RPE cells into one eye of two patients with two different forms of macular degeneration, dry age-related macular degeneration and advanced Stargardt macular dystrophy. The study [38] achieved good initial results as no signs of rejection, ectopic tissue formation, tumorigenicity or hyperproliferation were detected after 4 months of transplantation. Moreover, the authors reported increases in various functional endpoints [44,45,46] and detailed methods for generating a master cell bank of human embryonic cell stems for clinical application: in this case, human ESC differentiation resulted in greater than 99% pure RPE, with markers of pluripotency such as OCT4 and Homeobox protein NANOG. Despite encouraging results supporting the feasibility of using the human ESCs for cell-based retinal regenerative therapy, the potential of using ESCs in cell replacement therapy for the treatment of retinal diseases is still limited. The use of ESCs still poses ethical issues and the risk of immune rejection. In addition, naïve ESCs have been associated with teratoma formation after transplantation [47], and the efficiency of generation of functional RPE cells is too low and the timing too slow for the narrow window of effective therapy.

### 3.3. Induced Pluripotent Stem Cells (iPSCs)

The development of iPSCs provides several advantages as a source of retinal cells for transplantation, methods of drug testing and the development of models that can simulate human disease better than animal models [48]. The main study for generating iPSCs is authored by Takahashi et al. in 2007, where the creation of iPSCs from skin fibroblasts was induced with the viral transduction of four transcription factors—OCT4, SOX2, KLF4 and C-MYC [49,50]—that allowed mature cells to return to a pluripotent state similar to that seen in ESCs [51]. The preclinical efficacy of iPSCs must be proven before use in human trials. Studies of RPE-based disorders are the best candidates for iPSC modelling, given their accessibility through manual dissection and expansion on an assortment of substrates, behavior that mimics primary human prenatal in vitro, as well as the ease of monitoring the maturation state through distinct morphological features [52,53]. In 2012 Li et al. [54] published a pioneering study reporting methods to obtain and successfully transplant iPSC-derived RPE cells. Fibroblasts have been recovered from a skin biopsy and cocultured with mitomycin-C-treated PA6 feeder cells, which possess SIDA and promote RPE differentiation. The resulting iPSC-derived RPE cells were grafted subretinally into the subretinal space of a mouse possessing a mutation in a gene known to be responsible for certain types of retinitis pigmentosa [54], and they successfully restored retinal function, assessed by electroretinography. Other studies, such as the one by Maeda et al.’s group [55], have clarified the mechanism of action of iPSC-derived RPE cells as they produce the visual chromophore, 11-cis-retinal, and formed retinosomes in vitro. Additionally, iPSC-derived RPE cells were found to replace dysfunctional RPE cells on histological analysis. Further, it is no surprise that studies of retinitis pigmentosa in animal models using iPSC transplants have shown success in several studies [56]. All these encouraging data pose the basis for the treatment of retinal degenerative diseases, such as age-related macular degeneration, Stargardt disease and retinitis pigmentosa. Although phenotypically different, these diseases have shown significant promise in being treated with iPSCs, and they are the subject of many clinical trials using iPSCs to perform RPE transplantation [57]. Furthermore, many types of retinal cells, including those of the RPE, photoreceptors and ganglion cells, have been differentiated from iPSCs [58], allowing the development of iPSC-derived photoreceptors and ganglion cells. Finally, thanks to genetic similarities between humans and nonhuman primates, preclinical testing became possible with the development of iPSC cell lines from monkeys and their differentiation into RPE cells [59].

### 3.4. Generation of Retinal Organoids from Human Embryonic Stem Cells or Human Induced Pluripotent Stem Cells

Organoids are “mini-organs” generated from hESCs or hiPSCs. Organoids have been developed for several organs, including the liver, lung and pancreas. In a pioneering work, Eiraku and colleagues developed approaches to differentiate mouse ESCs into a 3D structure resembling a developing retina [60]. Moreover, their work showed that human stem cells could be differentiated into human retinal organoids, providing a platform to develop organoid-based methods for transplantation and therapies [61]. Additional studies showed that retinal organoids could be cultured into relatively advanced maturity stages in vitro [62,63]. Retinal organoids derived from hESCs or hiPSCs, especially hiPSCs, are affordable thanks to unlimited sources and few ethical issues, which makes the retinal organoid a popular tool for studying the pathogenesis of retinal diseases and graft treatments in vitro. Despite considerable progress, there are still many problems connected with retinal organoids [64]: first of all, the variations among batches; then, the methods to obtain cells suitable for transplantation from retinal organoids [64,65]; the safety and long-term survival of transplanted cells or tissues in vivo [65]; and finally, the efficiency of neural circuit formation in the host. For example, retinal organoid induction methods can be classified into three categories. The first category adapts a 2D to 3D process but does not go through an EBs stage [66,67]. According to this protocol, iPSCs are cultured to 70% confluence with a specific medium in culture dishes until self-forming neuroepithelial-like structures appear in about 4 weeks. The second category includes an EB stage [68] after ESCs are dissociated into small clumps and cultured in a specific medium. According to this protocol, the retina-like structures are dissected and collected in suspension for long-term culture until horseshoe-dome-shaped NR domains are formed (in about 4 weeks). The third category is the classic procedure reported by Sasai et al. [69] where ESCs are dissociated into single cells and quickly re-aggregated in a specific medium in low-cell-adhesion V-bottomed 96-well plates. Although retinal organoid technology has made a great leap forward in recent years, there are still many problems associated with the use of organoids, such as the high heterogeneity between cell lines and experimental results, the long culture times, the progressive degeneration of the inner cell layers and the purification of target cells.

### 3.5. Mesenchymal Stromal Cells (MSCs)

As discussed before, the use of ESCs or iPSCs is limited by the possibility of immune rejection, teratogenicity and ethical restrictions in the case of ESCs. At the same time, organoid technology still has problems to be solved before it can be applied in the clinic. For all these reasons, MSCs show great potential and could be a prospective tool for the treatment of retinal diseases [70,71]. The definition of MSCs is based on the phenotypic expression of a distinct set of cell surface markers as CD105, CD90, and CD73 but lacking CD79, CD45, CD34, CD19, CD14, CD11b and Human Leukocyte Antigen Class II (HLA-II) [72,73]. In addition, these cells can undergo in vitro tri-lineage differentiation into osteogenic, adipogenic and chondrogenic, as defined by the ISCT guideline for MSCs [73,74]. Another advantage of MSCs is that they can be easily isolated from many different tissues, exploiting their ability to adhere to plastic support. MSCs can be found abundantly in the adult tissues, such as bone marrow, adipose tissue and dental pulp, as well as in the fetal tissues and fluids, including the umbilical cord tissue, blood and amniotic fluid [75]. In addition to their wide distribution, MSCs are also known to possess minimal susceptibility to malignant transformation and are capable of avoiding immune cell recognition, hence providing a potential platform for allogeneic and autologous cell transplants [76]. There are many accumulative pre-clinical studies and clinical trials [77,78] demonstrating that the administration of MSCs has revealed significant restoration of the visual system; in particular, it has been shown that after an injection of MSCs into the vitreous body, the cells can survive for a long period and can protect retinal ganglion cell survival or stimulate axon regeneration after optic nerve crush [79]. Cellular reparative mechanisms of MSCs for retinal diseases have been discussed for a long time [71,80], basically involving four mechanisms:

*(a)*  Trans-differentiation;
*(b)*  Paracrine action for cell repair;
*(c)*  Immunoregulatory function;
*(d)*  Anti-angiogenic trophic action.

Moreover, there are multiple routes of administration to deliver MSCs into the posterior lining of the eye, including intravitreal, intraocular, epiretinal or subtenon injections, to treat patients affected with posterior eye diseases, including AMD, DR, retinal ischemia and RP. In particular, the injection of MSCs into the vitreous body of a rat model showed that the cells can survive for a long time and can protect retinal ganglion cell survival and stimulate axon regeneration [81]. Briefly, the possible cellular mechanisms utilized by MSCs in correcting ocular disorders will be discussed below.

**(a) Trans-differentiation**. MSCs, like stem cells, can differentiate into endodermal and ectodermal lineages thanks to their regenerative potential. Trans-differentiation is defined as a two-step differentiation process that involves the dedifferentiation of terminally differentiated cells and subsequent differentiation into specialized cells of a different lineage [82]. Regarding the ability of MSCs to differentiate into retina-like cells, including RPE cells, there are many extensive data: it has been reported that MSCs can differentiate into cornea-like cells [83,84], neurons [85] or various types of retinal cells [86], including RPE cells, which play an important role in the nourishment of photoreceptors [87]. An interesting review by Salehi et al. discusses the ability and the in vitro culture methods of the induction of MSCs to differentiate into retinal cells [88].

**(b) Paracrine action.** MSCs are strong producers of several growth and trophic factors. It is known that some of these factors are produced under inflammatory or mitogen stimuli while others are produced constitutively. The production of growth factors and especially their paracrine action are described as one of the main mechanisms of the therapeutic action of MSCs. In particular, it has been shown that the following growth factors can contribute to retinal regeneration: NGF, HGF, IGF-1, FGF, PEGF, GDNF, PDGF, EGF, angiopoietin-1, erythropoietin, VEGF and TGF-β [89,90]. The mechanism of action is the neurotrophic effect on the recipient neural cells, promoting cell survival, differentiation, axonal outgrowth and cell attachment and inhibiting neural cell apoptosis [91,92].

**(c) Immunoregulatory function.** It has been known for many years that MSCs exert a strong immunosuppressive activity against several cells of the immune system, such as T and B cells (inhibition of proliferation) and NK cells (inhibition of cytotoxic activity). The mechanisms behind this immunomodulatory potential include direct cell-to-cell contact, production of various immunomodulatory molecules, inhibition of antigen-presenting cells or the activation of regulatory T cells (Tregs). [93]. MSCs express numerous molecules that contribute to immunosuppression, such as the IDO, Cox-2, TSG-6, PDL-1 or Fas-L molecules. Furthermore, MSCs produce several cytokines that can negatively influence immune reactions, such as IL-6 and TGF- β. Dysregulation of the intraocular immune system is a pathological condition commonly manifested in AMD, glaucoma, diabetic retinopathy and uveitis, and it is represented by a profound release of pro-inflammatory cytokines, chemokines and MMPs, which progressively results in the loss of endothelium tight-junction proteins, destruction and leakage of BRB; hence, it facilitates the infiltration of immune cells. Thanks to their immunoregulatory properties, MSCs can modulate both the innate and adaptive immune responses and suppress immunoreactivity in eye disease [94].

**(d) Anti-angiogenic trophic action**. As a matter of fact, angiogenesis that leads to the formation of new blood vessels from pre-existing vessels is a process that has been recognized in several types of ocular disorders, including diabetic retinopathy, retinopathy of prematurity, retinal vein occlusion, AMD and glaucoma. In this context, MSCs show a strong therapeutic potential in the restoration of ocular neovascularization through the suppression of VEGF, MMP-9 and TLRs thanks to the downregulation of pro-inflammatory cytokine production [95]. As described before, MSCs secrete a wide range of growth factors and cytokines with proteolytic and angiogenic proprieties, such as VEGF, bFGF, TGF- 1, cathepsin, SDF-1 and PAI-1, in response to tissue repair. The interesting study by Gao et al. [96] shows how MSCs express secretory proteins under the pressure of pathological neovascularization in the eye. Moreover, several further studies conducted on mouse models support this thesis, as the transplantation of MSCs could stabilize neovascularization lesions and encourage corneal wound healing through angiogenesis modulation [97,98].

#### Additional Therapeutic Mechanisms of MSCs: MSC-EVs and Mitochondrial Transfer

In addition to the ability of MSCs to produce several growths and immunoregulatory or neurotrophic factors, MSCs release various types of EVs. These MSC-EVs or exosomes are secreted, bilipid-layered, nano-dimensional microvesicles that encapsulate functional molecules, such as proteins, lipids and miRNAs, and can provide important therapeutic effects, including the support of cell survival [99] and the protection of retinal ganglion cell function [100]. Furthermore, MSCs’ mitochondrial transfer represents an additional mechanism supporting anti-inflammatory conditions and cell survival [101]. According to this therapeutic action, mitochondrial transfer therapy could be improved in all those retinal diseases in which mitochondrial dysfunction has been documented.

## 4. Experimental Models and Clinical Trials

Inherited Retinal Dystrophies are currently a challenging area for new therapeutic approaches because of the lack of effective authorized treatment. In recent years, gene therapy trials and the approval of the first gene therapy for RPE65-related retinal degeneration changed the vision of the ineluctability of these devastating disorders. Cell-based therapies represent the new era of regenerative medicine, and many studies are emerging to define the best treatment options. Since this new technology, even if the first clinical studies started more than 10 years ago, is still in its infancy, many issues have arisen during preclinical and clinical approaches.

A common characteristic of retinal dystrophies is the death of the specialized retinal cells and the degeneration of the photoreceptors. Due to these characteristics, the treatment options for retinal dystrophies have been very limited [71]. Talking about experimental models, the literature reports several options: for example, the spontaneous or genetically induced degeneration of the photoreceptors by the administration of chemicals is one of the experimental models (natural or transgenic) of RP [102,103]. As an alternative to animal models, over the years, cell-based models have also been developed. For example, the primary culture of hfRPE or the immortalized ARPE-19 cell line are widely used as models for AMD research, thanks to their similar function and metabolic activity to native RPE. [104]. Concerning clinical trials, after the first futuristic transplantation of fetal human RPE cells performed by Algvere et al. in 1997, several initial clinical trials based on sCMTPs have started. In particular, the subretinal transplantation of hESC-derived RPE has been used in a few clinical trials for the treatment of Stargardt disease. Clinical trials in this field are usually very complex and lengthy, e.g., the first one started in (NCT01345006) April 2011 and ended in 2021 (Phase I/II). Thirteen patients were enrolled and sorted into different cohorts (each cohort received a different number of MA09- hESC-RPE cells—from 50,000 to 150,000). Another interesting study was performed by Schwartz et al. in 2015 [46], where some clinical data have been described after the transplantation of human embryonic stem cell-derived retinal pigment epithelium. Schwartz et al. found no evidence of adverse proliferation, rejection or serious ocular or systemic safety issues related to the transplanted tissue. Transplanted patients were followed for a median of 22 months using serial systemic, ophthalmic and imaging examinations. The BCVA in treated eyes and vision-related quality-of-life measures for general and peripheral vision improved 22 months after transplantation. The results of this study provide evidence of the medium-term to long-term safety, graft survival and possible biological activity of pluripotent stem cell progeny in individuals with any disease. Encouraged by these outcomes, in November 2011, a more comprehensive investigation of hRPE-ESC transplantation with a higher dose of hESC-RPE cells (50,000–200,000 cells) was conducted in the United Kingdom (NCT01469832) and concluded in 2021. They enrolled 12 patients who received hESC-RPE cells in the subretinal area; no evidence of uncontrolled proliferation or inflammatory responses was found, and improvement of visual acuity at 12 months of follow-up was not observed. In China, there is another ongoing study (NCT02749734) on the subretinal transplantation of hESC-RPE in patients affected by STGD, and in Poland, another one (NCT03772938) uses stem/progenitor cells transplantation. A particularly promising type of cell for the treatment of IRDs are MSCs, thanks to some peculiarities that distinguish them from all other therapeutic approaches. As discussed before, one of the main features of MSCs is the easy isolation from several tissues. Moreover, MSCs are known to have potent immunoregulatory activity and antiapoptotic properties that, when combined, can protect retinal cells from death and degeneration. Another characteristic is their ability to differentiate into various cell types, including the cells of the retina, contributing to the retina’s regeneration. Finally, MSCs produce a plethora of growth and neurotrophic factors, supporting the survival and growth of retinal cells. As a matter of fact, several clinical trials [71] showed very encouraging data, both for the safety of administration and efficacy of the treatment, as the administration of MSCs is not associated with complications or rejection and is associated with an improvement in visual function. Moreover, different types of retinal diseases, such as AMD, DR, RP, glaucoma, inherited retinal dystrophy, optic nerve diseases or macular holes, have tested the therapeutic effect of MSC transplantation from different sources, such as bone marrow or adipose tissue [105,106,107,108,109], with encouraging results. Finally, not only cells but also the conditioned medium obtained from cultured MSCs or the exosomes prepared by the ultracentrifugation have been experimented in clinical trials, providing encouraging effects [110]. 

Table 1 shows selected examples of clinical trials using sCTMPs for IRDS.

## 5. Summary

Advanced therapies are promising avenues for a new approach to changing the natural history of inherited retinal dystrophies. In the pediatric age group, the consequence of these devastating disorders represents a social burden that cannot be overlooked, and the beginning of treating rare eye diseases is quickly changing the perception of what science can do for these patients. Gene-therapy trials and the recent development of the first gene therapy for RPE65 demonstrated the possibility of treating rare diseases. Unfortunately, one of the limits in gene therapy is represented by the need for a vital target tissue since gene editing is useless if the target cells are absent or the retina is in an advanced stage of degeneration; this approach largely benefits from the early approach in treating progressive disorders when the retinal viable cells are sufficiently preserved to restore or maintain visual function. Advanced cell therapy medicinal products overcome this limit since their action does not necessarily need a preserved anatomy; although integrating retinal cells in such a complex circuitry as the retina represents a major challenge, the advancement in molecular biotechnology is demonstrating encouraging perspectives.

Cell therapy allows a wider range of opportunities, spanning from the use of ESCs—more difficult mainly for ethical aspects—to iPSCs—a simpler way with fewer ethical issues—to obtain a large amount of reprogrammed, undifferentiated cells. A particular mention has to be made of MSCs because of their different origin and properties; while iPSCs and ESCs can be a valuable way to obtain neuroretinal precursors and differentiate them in photoreceptors and other retinal cells [111,112,113,114,115], MSCs’ role seems, in prevalence, related to the support of the existing architecture by the secretion of neurotrophic factors or by integration within the damaged retina. Actually, the nature of mesenchymal tissue in embryology is mainly, even if not exclusively, related to the development of connective tissue, and the consequent supporting role is a natural prerogative of these cells. Recent studies demonstrated that MSCs, even if not integrated into the retinal architecture, can support retinal metabolism in degenerative retinal diseases; interestingly, the supportive role can also be obtained by the isolated secretion of products of MSCs, namely EVs, that have demonstrated to be useful in slowing down retinal degeneration. Due to broadening choices in therapeutical perspectives, one can argue that multiple approaches could also be a viable option in the near future since different treatment modalities can offer a complementary mode of effectiveness. In fact, retinal tissues can be variably involved during the pathological, degenerative process, and it is intuitive that, generally speaking, the earlier the onset of the retinal dystrophy, the more conserved the related retinal structure could be, versus late-stage diseases in adulthood, when the degenerative process can often induce a severe loss of retinal vital cells. The consequence of this consideration is that, in the early phase, the main goal could be represented by a supportive role of ATMPs (i.e., EVs, MSCs, etc.) aimed to mainly preserve retinal anatomy. In parallel, the potential theoretical association of gene-editing techniques could aim to reduce, at an early age, the need for the more complex approach with regeneration, probably necessary in late-stage disease when viable retinal tissue is often lacking and needs to be substituted by a new, healthy donor or engineered tissue. We think this new era of treating rare diseases could also point to combining more complex approaches with the regeneration of the atrophic area with new cell lines and maintenance of the residual environment with supportive cells or other medicinal products.

## Figures and Tables

**Table 1:** Selected examples of clinical trials using sCTMPs for IRDS.

Title/NCT Number	Retinal Disease	Cells for Treatment	Method of Administration	Study Type	Results
Intravitreal Autologous Bone Marrow CD34+ Cell Therapy for Ischemic and Degenerative Retinal Disorders	AMD, RP, retinal vascular occlusion	Autologous BM-MSC	Intravitreal	Phase I	No severe safety issues associated with treatment
Umbilical cord derived mesenchymal stem cell implantation in retinitis pigmentosa	RP	Umbilical cord- derived MSC	Suprachoroidal	Phase III	Improvements in best-corrected visual acuity, electroretinography and visual field
Subretinal adipose tissue-derived mesenchymal stem cell implantation in advanced stage retinitis pigmentosa	RP	A-MSC	Subretinal	Phase I	Minor ocular complications, no severe safety issues associated with the treatment
Management of Retinitis Pigmentosa by Mesenchymal Stem Cells by Wharton’s Jelly Derived Mesenchymal Stem Cells NCT04224207	RP, inherited retinal dystrophy	Wharton’s Jelly-derived MSC	Subtenons	Phase III	Improvement in visual acuity and in outer retinal thickness
Stem Cells Therapy in Degenerative Diseases of the Retina NCT03772938	Retinal degeneration, RP, Stargardt disease 1, AMD	Autologous bone marrow-derived stem/progenitor cells	Intravitreal	Phase I	No severe safety issues associated with the treatment
Clinical Study of Subretinal Transplantation of Human Embryo Stem Cell Derived Retinal Pigment Epitheliums in Treatment of Macular Degeneration Diseases NCT02749734	Macular degeneration, Stargardt macular dystrophy	Human embryo stem cell-derived retinal pigment epitheliums (hESC-RPEs)	Subretinal	Phase I Phase II	Sustained improvements in visual acuity and evidence of cellular engraftment
Clinical Trial of Autologous Intravitreal Bone-marrow CD34+ Stem Cells for Retinopathy NCT01736059	Non-exudative age-related macular degeneration, diabetic retinopathy, retina vein occlusion, RP, hereditary macular degeneration	Bone marrow CD34+ stem cells	Intravitreal	Phase I	Therapy is well-tolerated with no intraocular inflammation or hyperproliferation; best-corrected visual acuity and full-field ERG showed no worsening after 6 months
Sub-retinal Transplantation of hESC Derived RPE (MA09-hRPE) Cells in Patients with Stargardt’s Macular Dystrophy NCT01345006	Stargardt Macular Dystrophy	hESC-derived RPE (MA09-hRPE) cells	Subretinal	Phase I Phase II	No severe safety issues associated with the treatment

## Abbreviations

**A-MSCs**	adipose tissue-derived mesenchymal stem cell
**AMD**	age-related macular degeneration
**ATMP**	advanced therapy medicinal product
**ABCA4**	ATP-binding cassette transporter
**BM-MSCs**	bone marrow-derived mesenchymal stem cells
**BRB**	blood–retinal barrier
**COX-2**	cyclooxygenase-2
**DR**	diabetic retinopathy
**EBs**	embryoid bodies
**EGF**	epidermal growth factor
**ESCs**	embryonic stem cells
**EU**	European Union
**FAF**	fundus autofluorescence
**FDA**	Food and Drug Administration
**ffERG**	full-field electroretinogram
**FGF**	fibrocyte growth factor
**GCP**	good clinical practice
**GDNF**	glial cell-derived neurotrophic factor
**GMP**	good manufacturing practices
**hESC-RPE**	human embryonic stem cell-derived retinal pigmented epithelium
**hESCs**	human embryonic stem cells
**hfRPE**	human fetal retinal pigment epithelium
**HGF**	hepatocyte growth factor
**hiPSCs**	human induced pluripotent stem cells
**IDO**	indoleamine 2,3-deoxygenase
**IGF-1**	insulin-like growth factor-1
**IL-6**	interleukin-6
**iPSCs**	induced pluripotent stem cells
**IRD**	inherited retinal dystrophies
**ISCT**	International Society for Cellular Therapies
**KLF4**	Krüppel-like factor 4
**MMP-9**	matrix metalloproteinase 9
**MMPs**	matrix metalloproteinases
**MSC-EVs**	MSC-derived extracellular vesicles
**MSCs**	mesenchymal stromal cells
**NGF**	nerve growth factor
**NK**	natural killer
**OCT**	optical coherence tomography
**OCT4**	octamer-binding transcription factor 4
**PAI-1**	plasminogen activator inhibitor 1
**PDGF**	platelet-derived growth factor
**PDL-1**	programmed death-ligand 1
**PEGF**	pigment epithelium growth factor
**RP**	retinitis pigmentosa
**RPCs**	retinal progenitor cells
**RPE**	retinal pigment epithelium
**sCTMP**	somatic cell therapy medicinal product
**SDF-1**	stromal cell-derived factor 1
**SIDA**	stromal-derived inducing activity
**STGD**	Stargardt disease
**TGF- β**	transforming growth factor beta
**TLRs**	toll-like receptors
**TSG-6**	TNF-α stimulated gene 6 protein
**VEGF**	vascular endothelial growth factor
